# Barcoding success as a function of phylogenetic relatedness in *Viburnum*, a clade of woody angiosperms

**DOI:** 10.1186/1471-2148-12-73

**Published:** 2012-05-30

**Authors:** Wendy L Clement, Michael J Donoghue

**Affiliations:** 1 Department of Ecology and Evolutionary Biology, Yale University, P.O. Box 208105, New Haven, CT, 06520, USA

## Abstract

**Background:**

The chloroplast genes *mat*K and *rbc*L have been proposed as a “core” DNA barcode for identifying plant species. Published estimates of successful species identification using these loci (70-80%) may be inflated because they may have involved comparisons among distantly related species within target genera. To assess the ability of the proposed two-locus barcode to discriminate closely related species, we carried out a hierarchically structured set of comparisons within *Viburnum*, a clade of woody angiosperms containing ca. 170 species (some 70 of which are currently used in horticulture). For 112 *Viburnum* species, we evaluated *rbc*L *+ mat*K, as well as the chloroplast regions *rpl*32-*trn*L, *trn*H-*psb*A, *trn*K, and the nuclear ribosomal internal transcribed spacer region (nrITS).

**Results:**

At most, *rbc*L *+ mat*K could discriminate 53% of all *Viburnum* species, with only 18% of the comparisons having genetic distances >1%. When comparisons were progressively restricted to species within major *Viburnum* subclades, there was a significant decrease in both the discriminatory power and the genetic distances. *trn*H-*psb*A and nrITS show much higher levels of variation and potential discriminatory power, and their use in plant barcoding should be reconsidered. As barcoding has often been used to discriminate species within local areas, we also compared *Viburnum* species within two regions, Japan and Mexico and Central America. Greater success in discriminating among the Japanese species reflects the deeper evolutionary history of *Viburnum* in that area, as compared to the recent radiation of a single clade into the mountains of Latin America.

**Conclusions:**

We found very low levels of discrimination among closely related species of *Viburnum*, and low levels of variation in the proposed barcoding loci may limit success within other clades of long-lived woody plants. Inclusion of the supplementary barcodes *trn*H-*psb*A and nrITS increased discrimination rates but were often more effective alone rather than in combination with *rbc*L *+ mat*K. We surmise that the efficacy of barcoding in plants has often been overestimated because of the lack of comparisons among closely related species. Phylogenetic information must be incorporated to properly evaluate relatedness in assessing the utility of barcoding loci.

## Background

The use of a short fragment of DNA sequence to distinguish between species -- DNA barcoding -- promises to streamline species identification, thereby enabling scientific research (e.g., studies of community ecology) and practical applications (e.g., monitoring the movement of biological materials across borders). The ideal DNA barcode would be a single locus that could be universally amplified and sequenced for a broad range of taxa, be easily aligned over large phylogenetic distances, and provide sufficient variation to reliably distinguish closely related species. The zoological community has adopted cytochrome oxidase I (COI) as a DNA barcode that appears to generally fulfill these criteria. In contrast, the plant community has struggled to identify a single marker with these qualities [1,2] and botanists have favored the use of a multilocus barcode [3-5]. Specifically, the Plant Working Group of the Consortium for Barcodes of Life has proposed the combined use of short segments of the chloroplast genes *mat*K and *rbc*L as a “core” plant barcode [5]. However, in view of the fact that *mat*K and *rbc*L have not been considered the best choices in a number of individual studies ([2,6-9] but see also [10,11]), the use of supplementary, typically more variable barcodes, such as *trn*H-*psb*A and the nuclear ribosomal internal transcribed spacer regions (nrITS), has been suggested as a means of increasing the efficacy of the *rbc*L *+ mat*K barcode [12].

**Figure 1:**
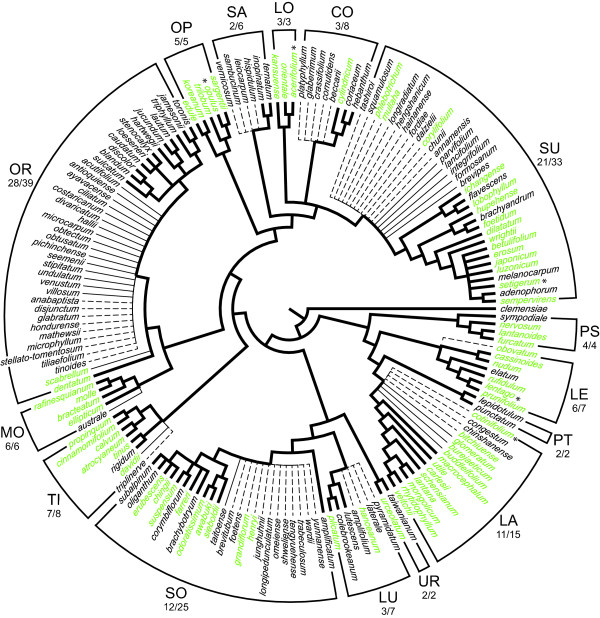
**Phylogenetic representation of the 170 currently recognized species of*Viburnum***. Branches in bold represent the 90 species and evolutionary relationships recovered in Clement and Donoghue [15]. Thin branches are those species added to the study of *Viburnum* in this paper, and dashed branches are *Viburnum* species that have yet to be sampled. These species are placed at the base of the named clade to which they are expected to belong based on previous taxonomic and morphological studies. Species names in green are those that are currently used in the horticultural industry, and species marked by an asterisk were sampled in Fazekas et al. [4] and in CBOL Plant Working Group [5]. Informal clade names (following [15]), and the number species sampled in this study followed by the total number of species assigned to the clade, are provided. Abbreviations are as follow: CO-*Coriacea*, LA-*Lantana*, LE-*Lentago*, LO-*Lobata*, LU-*Lutescentia*, MO-*Mollodontotinus*, OP-*Opulus*, OR-*Oreinodontotinus*, PS-*Pseudotinus*, PT-*Punctata*, SA-*Sambucina*, SO-*Solenotinus*, SU-*Succodontotinus*, TI-*Tinus*, UR-*Urceolatum*.

In the search for a plant barcode, universality and ease of amplification and sequencing have been prioritized [4,5,13], and these criteria played a major role in the choice of *rbc*L *+ mat*K [5]. The discriminatory power of *rbc*L *+ mat*K has been evaluated in a number of studies, but the effects of taxon sampling in such studies requires further analysis. In several studies that have presented comparisons that widely span the angiosperms, it has been calculated that *rbc*L *+ mat*K are able to distinguish 70-80% of the species [3-5,14]. As a proxy for comparing closely related species, some of these studies have included two or more species from within a number of plant genera, but phylogenetic trees were not specifically used to gauge the relatedness of the species sampled. This is problematical. For example, when placed in a phylogenetic context (Figure 1), the five species of the genus *Viburnum* (Adoxaceae, Dipsacales) that have been included in such comparisons [4,5] turn out to represent widely separated clades that have been diverging from one another for tens of millions of years. Comparing only these species may overestimate the ability to distinguish among closely related species using the proposed markers. Generally, because genera come in many sizes and ages, the random sampling of selected species within a genus does not ensure that these species are actually very closely related to one another. Direct phylogenetic information is necessary to determine how closely or distantly related the species are.

The success of barcoding also depends on the analytical methods employed. So-called character-based approaches [16] can differentiate plant species based on one or a few variable base pairs, while more commonly used methods based on genetic distances (e.g., using a predetermined cut-off of 1%) or tree-based approaches may require greater amounts of genetic variation [9]. Here too, it is important to test such methods on species whose relatedness has been inferred phylogenetically. To establish meaningful barcoding guidelines and standards, it ultimately will be essential to carry out comparisons of both markers and analytical methods within a well-defined phylogenetic framework.

Some barcoding applications, such as inventories of biodiversity hotspots [17], require the differentiation of species only within a given geographic area, and comparisons within regions have generally reported higher species discrimination rates using plant barcodes ([12,18,19], but see [20]). For example, Kress et al. [18] were able to discriminate 98% of the species in barcoding the plants on Barro Colorado Island in Panama; the only problems were within genera with more than one species on the Island, such as *FicusInga*, and *Piper*. Such results may reflect a general pattern, namely that very closely related plant species seldom grow sympatrically. However, as some evolutionary circumstances can yield such sympatry (e.g., polyploidy speciation), the efficacy of community-level or regional barcoding efforts also needs to be evaluated in a phylogenetic context. In general, we would expect better discrimination when the several members of particular genera within an area represent relatively distantly related clades.

Here we evaluate the discriminatory power of potential plant barcodes within the context of a phylogeny for the woody flowering plant clade *Viburnum* (Adoxaceae, Dipsacales). This clade contains approximately 170 species (Figure 1) and is of great interest to the horticultural community as more than 70 of these species (and various artificial hybrids) are currently in cultivation ([21]; Figure 1). The ability to distinguish closely related *Viburnum* species using barcodes would be extremely useful in identifying horticultural material and in monitoring the movement of these economically important plants (as cuttings or seeds) around the globe.

*Viburnum* naturally occupies the temperate regions of North America and Eurasia and extends into the montane forests of Latin America and into tropical habitats in Southeast Asia. Most *Viburnum* species are diploids with 2N = 18 [22,23]. Homoploid speciation has been postulated in a few specific instances [24-26], though evidence for this is still limited. Allopolyploidy appears to have occurred several times [23,25]. The New World *Oreinodontotinus* clade is characterized by chromosome numbers of 36 and, occasionally, of 72 [22,23,27]. An aneuploidy reduction to 2N = 16 characterizes the Asian *Solenotinus* clade, within which chromosome numbers of 32 and 64 are also found [23]. Hybridization is possible between members of the different section-level clades [22], but it is not especially common in the wild, and hybrid swarms and introgression have seldom been documented and are associated with recent human disturbance [27].

Although the species-level taxonomy of *Viburnum* is currently under review, many steps have recently been taken to confirm the number of species that exist in the wild. Approximately 894 *Viburnum* species names appear in IPNI (http://www.ipni.org), Tropicos (http://www.tropicos.org) and The Plant List (http://www.theplantlist.org). More than a decade ago, Malécot and Donoghue (unpublished) reduced this list to 229 recognized species (the remaining names being placed in synonymy). In light of recent regional studies and other ongoing assessments, this list has been further refined and we now recognize ca. 170 species (Figure 1). Additionally, a series of recent phylogenetic studies has confidently identified the major clades within *Viburnum* and their relationships to one another [15,25,26,28]. These studies provide a solid framework within which to evaluate the power of barcode markers and methods to discriminate species globally, or within particular geographic regions, as a function of their degree of relatedness. Specifically, we focus on a set of hierarchically structured comparisons within *Viburnum* using the *rbc*L *+ mat*K core barcode, as well as three other chloroplast markers (*rpl*32-*trn*L, *trn*K, and *trn*H-*psb*A) and the nrITS region. *trn*H-*psb*A was once a contender as the plant DNA barcode [3,5,29], and the utility of ITS2 has recently been highlighted as an alternative to *rbc*L *+ mat*K [30-32]. In addition to making comparisons within and across all of *Viburnum*, we also evaluate the performance of these markers in a regional context, focusing especially on *Viburnum* species within Japan and within Mexico and Central America.

## Methods

### Species sampling

We obtained sequences from all of the 90 species used in our most recent phylogenetic study [15], with the exception of *V. lepidotulum*, from which we were able to obtain too few sequences. To this sample we added data for 28 previously unsequenced *Viburnum* species. As explained below, we lumped several pairs of previously separated species so as not to underestimate the discriminatory power of the plant barcodes. In total, we analyzed 112 species, 40 of which were represented by two to six individuals. Material for the newly acquired accessions was obtained from herbarium specimens from the Harvard University Herbaria (HUH), the Field Museum (F), the Missouri Botanical Garden (MO), the New York Botanical Garden (NY), and our own collections in silica gel with corresponding voucher specimens in the Yale University Herbarium (YU). Voucher information and Genbank accession numbers are provided in Additional file 1.

As they were in part designed to test the relationships of proposed “segregate” species, our previous phylogenetic studies included representatives of several potential *Viburnum* species that are not presently considered to be distinct in recent regional taxonomic treatments. For present purposes we wanted to reduce the number of species in these cases so as not to bias the barcoding results by artificially reducing genetic distances. Specifically, we lumped *V. awabuki* with *V. odoratissimum*[33], *V. calvum* with *V. atrocyaneum*[34], *V. scabrellum* with *V. dentatum*[35], *V. taiwanianum* with *V. urceolatum*[33], and *V. veitchii* with *V. glomeratum*[34]. In several instances, however, we did not reduce species complexes as proposed in some regional floras based on our own conflicting geographic or molecular evidence. Thus, we maintained *V. australe* and *V. affine* as distinct from *V. rafinesquianum* on the basis of their geographic ranges. Also, in view of the results of Clement and Donoghue [15], we treated *V. adenophorumV. flavescensV. hupehense*, and *V. lobophyllum* as distinct from *V. betulifolium* (contra [34]). Similarly, we recognized *V. bracteatum* as distinct from *V. molle*, and *V. cylindricum* as distinct from *V. coriaceum*.

### DNA extraction and data collection

Total genomic DNA was extracted from herbarium and silica dried specimens using a Qiagen DNeasy kit (Valencia, CA). The initial step of the extraction protocol was modified for herbarium tissue by adding B-mercaptoethanol and proteinase K to ground leaf tissue and shaken for 12-24 hours at 42°C [36].

Amplification and sequencing protocols for *mat*K, *trn*H-*psb*A, *rpl*32-*trn*L, *trn*K, and nrITS followed Clement and Donoghue [15]. The barcoding region of *rbc*L was obtained from previously sequenced taxa by truncating the sequences to match the proposed barcoding region. In instances where we were unable to sequence the entire *rbc*L gene region, we followed the *rbc*L barcoding protocol [5] using *rbc*La_f [3] and *rbc*La_rev [5] primers.

PCR products were sequenced in forward and reverse directions using the amplification primers at either the DNA Analysis Facility on Science Hill or the Keck DNA Sequencing Facility at Yale University. Sequences were assembled using Sequencher 4.10.1 (Gene Codes Corp.) and aligned using Muscle 3.6 [37]. Gene region alignments were manually reviewed and edited.

### Phylogenetic analysis

With 28 species new to the study of the *Viburnum* phylogeny, we conducted a phylogenetic analysis including one representative of all 112 species and the six genes examined in this study (Additional file 1). The data were separated into two partitions, one containing all chloroplast gene regions and the second containing nrITS. Models for each partition were selected using MrModeltest [38]. Phylogenetic analyses were performed with MrBayes v3.1.2 [39], with 30 million generations using six chains, sampling the posterior distribution every 1,000 generations. Plots of the likelihood and model parameters were examined in Tracer 1.5 [40] to assess convergence and determine an appropriate burnin.

### Barcode evaluation and species identification

We evaluated six candidate plant barcoding markers, including five chloroplast regions and nrITS. First, each gene region was evaluated independently. Then, we concatenated and evaluated *rbc*L and *mat*K together, as this is the core plant DNA barcode proposed by the CBOL Plant Working Group [5]. Lastly, we concatenated a third gene region (supplementary barcode) to this core barcode. Specifically, we evaluated the discriminatory power of *rbc*L + *mat*K + *trn*H-*psb*A and of *rbc*L + *mat*K + nrITS. Because the number of accessions per species varied, calculations involving interspecific comparisons were obtained from a data set that included only one representative accession per species (Additional file 1). Intraspecific comparisons were made separately.

We evaluated potential barcodes in three ways. First, we identified the number of unique sequences (i.e., haplotypes) within each data set using TCS [41], which provided an absolute maximum number of species that could be identified with the data. With this approach, successful discrimination of two species could entail a difference of just one base pair. Then, the number of unique sequences was divided by the number of species included in the dataset to obtain an estimate of the maximum percentage of species that could be discriminated by the data. Second, we calculated genetic distances under a Kimura 2 parameter (K2P) model using PAUP 4b10 [42] for both intra- and interspecific comparisons. We did not include the same number of accessions per species, and not all species were represented by more than one accession. To control for this, we averaged the intraspecific variation within each species to prevent artificially increasing or decreasing the overall levels of interspecific variation detected in the data. Histograms were compiled using R version 2.13.0 [43] to examine the variation in the data and to compare intra- and interspecific genetic distances. Third, using the genetic distances generated from pair wise comparisons among all species in the data set, we report the percentage of comparisons with genetic distances that exceed 1% and 2%. We recognize that any such cutoff is arbitrary, but these cutoffs appear commonly in the literature and allows a comparison to results from less inclusive clades of *Viburnum* as described below.

### Hierarchical evaluation of barcode performance

To explore the discriminatory power of barcoding regions in an evolutionary framework, we used our *Viburnum* phylogeny to inform a set of comparisons. Specifically, we focused on the four largest named clades within *Viburnum*: *LantanaOreinodontotinusSolenotinus*, and *Succodontotinus*[15,26]. We compiled the data described above for each of these four clades separately: 11 of the ~15 species of *Lantana*, 28/~39 species of *Oreinodontotinus*, 12/~25 species of *Solenotinus*, and 21/~33 species of *Succodontotinus*.

### Barcode evaluation using regional samples

To explore the discriminatory power of the various barcodes within more restricted geographical areas, we focused on two regions: Japan and Mexico and Central America. Our data include 14 of the 16 species described from Japan [33], and all 17 species described from Mexico and Central America [27]. We compiled the standard nine datasets for each of the two geographical regions and analyzed the data as described above.

## Results

### Discriminatory power across *Viburnum*

Information on the number of species sampled, total aligned sequence length, and number of variable characters for each gene region and combination of gene regions is given in Table 1. The number of identical sequences in the datasets is also shown in Table 1. For this calculation, gaps were treated as missing data, so the differences between sequences were based only on point mutations (nucleotide substitutions). When gaps were coded as a 5^th^ state, the number of unique sequences increased for all gene regions except *mat*K and *rbc*L (Table 1). However, using gaps as traits is difficult because the occurrence of gaps can change depending on taxon sampling; gaps could prove useful once all species of *Viburnum* have been properly sampled.

**Table 1:** ***Viburnum *interspecific comparisons for barcode gene regions**

						**K2P Genetic Distances**			
**Gene Region**	**Species**	**Aligned Length**	**Variable characters**	**Unique sequences**	**% Max ID rate**	**Max**	**Mean (SD)**	**> 1%**	**> 2%**
*mat*K	98	725	62	38	38.78	0.0255	0.0087 (0.0047)	34.36	0.95
*rbc*L	103	491	18	20	19.42	0.0187	0.0058 (0.0047)	24.77	0
*rpl*32-*trn*L	97	942	119	61 (70)	62.89	0.0352	0.0152 (0.0073)	78.95	27.32
*trn*H-*psb*A	108	491	103	54 (79)	50.00	0.0597	0.0184 (0.0098)	84.94	41.64
*trn*K	97	1068	90	48 (52)	49.48	0.0211	0.0108 (0.0053)	64.37	1.50
nrITS	105	628	202	94 (97)	89.52	0.1117	0.0528 (0.02)	94.63	89.80
*rbc*L + *mat*K	94	1216	79	50	53.19	0.0184	0.0074 (0.0033)	18.23	0
*rbc*L + *mat*K + *trn*H-*psb*A	91	1707	175	67	73.63	0.0231	0.0100 (0.0041)	67.94	0.46
*rbc*L + *mat*K + nrITS	88	1844	261	86	97.73	0.0396	0.0219 (0.0078)	88.71	72.52

For each gene region, the number of species analyzed, the aligned length of the gene region, the number of variable characters, the number of unique sequences with gaps treated as missing data (number with gaps treated as a fifth state), and the maximum number of species that can be identified by the data (Max ID rate = Identical sequences/total number of species) are reported. Also provided are summary statistics of genetic distances using a Kimura 2-parameter (K2P) model and include: maximum genetic distance (max), mean interspecific distance (mean) with standard deviation (SD), and the proportion of comparisons of genetic distances greater than 1% (>1%) and greater than 2% (>2%). See Additional file 1 for details on species sampling.

The number of identical sequences was used to calculate a maximum identification proportion (Max ID rate; Table 1). In this case, two species need differ by only a single base pair to be considered successfully differentiated. Applying this approach to the *mat*K and *rbc*L data, we were only able to identify 39% and 19% of the species sampled, respectively, and just over 50% when the two regions were combined (Table 1). The other chloroplast regions sampled yielded slightly higher proportions (~49-63% of species differentiated). nrITS was the most variable gene region and by itself could discriminate 90% of the species sampled.

**Figure 2:**
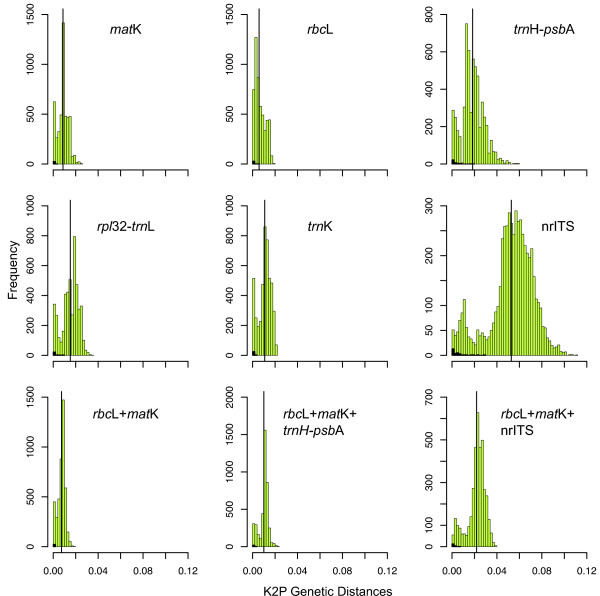
**Intra- and interspecific genetic distances of barcoding genes across*Viburnum***. Histograms showing intraspecific (black bars) and interspecific (green bars) genetic distances calculated using a K2P model of sequence evolution for each gene region or combination of gene regions. Each histogram contains the name of the gene region or regions sampled and a black vertical line indicating the mean genetic distance for the gene region(s).

Intra- and interspecific genetic distances were calculated as a second approach to evaluating discriminatory power (Figure 2; Tables 1 and 2). Mean interspecific genetic distances for *mat*K and *rbc*L were 0.0087 and 0.0058, respectively, and still less than 1% when combined. All of the other barcoding regions evaluated have mean genetic distances greater than 1% (Figure 2; Table 1). The mean intraspecific variation for each barcode was quite low with average comparisons for regions of 0.58% or less (Table 2). Even with our limited sampling of intraspecific variation, we observed complete overlap of the distributions of intraspecific and interspecific variation (Figure 2), so there was no natural “barcoding gap” [44] to use as a cut-off for distinguishing species. Minimum genetic distances for both intra- and interspecific comparisons were zero, and for most gene regions there were a significant number of comparisons with a genetic distance of zero. In the absence of a clear gap, we calculated discriminatory power using 1% and 2% differences. At the level of 1%, *rbc*L *+ mat*K distinguished 18% of the species; less than 1% of species comparisons differed by more than 2% (Table 1). This indicates that the majority of the unique sequences identified differed at very few nucleotide sites.

**Table 2:** ***Viburnum *intraspecific comparisons based on Kimura 2-Parameter (K2P) genetic distances**

**Gene Region**	**Accessions**	**Species**	**Max**	**Mean (SD)**
*mat*K	75	26	0.0028	0.0002 (0.0006)
*rbc*L	92	31	0.0041	0.0004 (0.0009)
*rpl*32-*trn*L	94	32	0.0084	0.0020 (0.0027)
*trn*H-*psb*A	109	39	0.0187	0.0029 (0.0043)
*trn*K	82	28	0.0038	0.0008 (0.0011)
nrITS	90	32	0.0295	0.0058 (0.0073)
*rbc*L + *mat*K	68	23	0.0017	0.0002 (0.0004)
*rbc*L + *mat*K + *trn*H-*psb*A	64	22	0.0025	0.0006 (0.0008)
*rbc*L + *mat*K + nrITS	57	19	0.0075	0.0018 (0.0019)

### Subclade analyses

**Figure 3:**
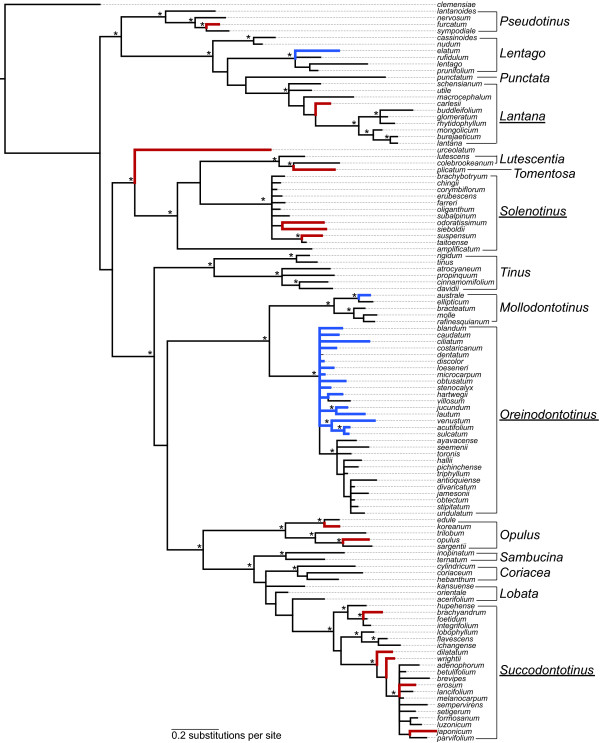
**Phylogeny of the 112 species of*Viburnum*sampled in this study**. Presented is the Bayesian majority rule consensus tree of the combined chloroplast and nrITS data. Asterisks above the branches indicate posterior probabilities greater than 0.95. The named clades within *Viburnum* proposed by Winkworth and Donoghue [26] and Clement and Donoghue [15] are shown to the right, and the underlined clade names are those used in the hierarchical comparisons (see text). Thick red and blue branches mark *Viburnum* species that occur in Japan and in Mexico and Central America, respectively. The chloroplast and nrITS partitions were each analyzed under a GTR + I + G model of sequence evolution. Resulting tree statistics and rate parameters are as follows: -lnL = 14052.08; chloroplast partition – rate matrix = 0.2398, 0.1810, 0.0819, 0.0903, 0.1738, 0.2331, basepair frequencies = 0.3224, 0.1643, 0.1700, 0.3435, G = 0.0602, I = 0.7412; nrITS partition – rate matrix = 0.0662, 0.2027, 0.0490, 0.0341, 0.6076, 0.0405, base pair frequencies = 0.1913, 0.3203, 0.2880, 0.1989, G = 0.7646, I = 0.4152.

The Bayesian analysis of all six genes sampled in this study (Figure 3) recovered all of the major clades identified in Clement and Donoghue [15] with the exception that here the three species of *Lobata* do not form a clade (Figure 3). In some instances, support for previously recognized clades was diminished, but this is likely due to the reduction in the genes sampling: six genes and 4,345 bp as compared to ten genes and 9,552 bp in Clement and Donoghue [15].

**Figure 4:**
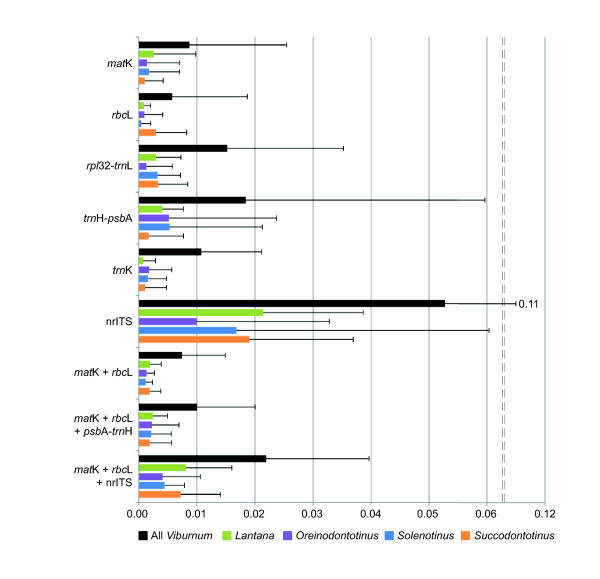
**Genetic variation within*Viburnum*subclades**. Bar graph showing the mean genetic distances calculated using all included *Viburnum* species and genetic distances calculated using the included species from four clades nested within *Viburnum*. The maximum genetic distance of each gene region or regions is indicated by a thin bar extending from the mean.

As expected, comparisons within the *Lantana*, *Oreinodontotinus*, *Solenotinus*, and *Succodontotinus* clades (Figure 3) showed a significant decrease in the level of genetic variation relative to comparisons made across all of *Viburnum* (Figure 4). For each gene region or combination of regions, the genetic variation decreased by more than 50% (Figure 4; Additional file 2). With the exception of nrITS alone and *rbc*L *+ mat*K + nrITS, none of the mean genetic distances exceeded 1% (Figure 4).

### Regional comparisons

Mean genetic distances among the Mexican and Central American species were very low (Table 3) and similar to results for the *Oreinodontotinus* clade that includes all but two of the species from this region (*V. elatum* of the *Lentago* clade; *V. australe* of the *Mollodontotinus* clade). Using the proposed barcoding markers, a maximum of 40% of the species could be identified and the average genetic distance among these species was only 0.1%. nrITS was the most variable locus, followed by *trn*H-*psb*A. In Japan, *rbc*L *+ mat*K discriminated many more species, and higher levels of genetic variation were observed for all of the markers (Table 3).

**Table 3:** **Summary of interspecific comparisons for regional *Viburnum *samples**

**Gene Region**	**Species**	**Aligned Length**	**Variable characters**	**Unique sequences**	**% Max ID rate**	**Max**	**Mean (SD)**	**> 1%**	**> 2%**
***Central America and Mexico (17 species)***									
*mat*K	14	719	10	3	21.29	0.0126	0.0022 (0.0044)	14.29	0
*rbc*L	12	491	3	4	33.33	0.0041	0.0015 (0.0018)	0	0
*rpl*32-*trn*L	15	876	22	8	53.33	0.0208	0.0038 (0.0057)	13.33	0.95
*trn*H-*psb*A	16	410	17	10 (12)	62.50	0.0265	0.0084 (0.0065)	35.83	8.33
*trn*K	12	1057	24	7 (8)	58.33	0.0202	0.0047 (0.0066)	16.67	6.06
nrITS	17	602	77	14 (15)	82.35	0.0829	0.0213 (0.0222)	56.62	33.09
*rbc*L + *mat*K	10	1210	5	4	40.00	0.0033	0.0010 (0.0012)	0	0
*rbc*L + *mat*K + *trn*H-*psb*A	11	1612	15	7 (9)	63.63	0.0070	0.0025 (0.0018)	0	0
*rbc*L + *mat*K + nrITS	11	1807	61	10	91.00	0.0232	0.0068 (0.0060)	20.00	3.63
***Japan (16 species)***									
*mat*K	13	725	24	10	76.92%	0.0155	0.0081 (0.0040)	32.05	0
*rbc*L	13	491	8	6	46.15%	0.0144	0.0065 (0.0048)	35.90	0
*rpl*32-*trn*L	13	873	46	11	84.62%	0.0266	0.0162 (0.0073)	76.92	35.90
*trn*H-*psb*A	13	439	41	10	76.92%	0.0477	0.0176 (0.0099)	85.90	34.62
*trn*K	14	1057	36	10	71.43%	0.0172	0.0093 (0.0043)	48.35	0
nrITS	13	608	104	13	100.00%	0.0914	0.0551 (0.0213)	97.44	89.74
*rbc*L + *mat*K	13	1216	32	11	84.62%	0.0125	0.0074 (0.0032)	12.82	0
*rbc*L + *mat*K + *trn*H-*psb*A	12	1654	65	10	83.33%	0.0157	0.0097 (0.0041)	71.21	0
*rbc*L + *mat*K + nrITS	12	1823	134	12	100.00%	0.0372	0.0230 (0.0089)	86.36	69.70

For the *Viburnum* species of Mexico and Central America and for Japan, the number of species analyzed, the aligned sequence length, the number of variable characters, the number of unique sequences with gaps treated as missing data (and with gaps treated as a fifth state), and the maximum number of species that can be identified by the data (Max ID rate = Identical sequences/total number of species) are reported. Summary statistics of genetic distances using a Kimura 2-parameter (K2P) model include: maximum genetic distance (max), mean interspecific distance (mean) with standard deviation (SD), and the proportion of comparisons of genetic distances greater than 1% (>1%) and greater than 2% (>2%).

## Discussion

We sampled approximately two thirds of all *Viburnum* species (112 of 170 species) and were able to distinguish at most 53% of the species sampled using the proposed plant barcode, *rbc*L *+ mat*K, and a character-based method that accepts even single base differences between species (Table 1). Similar upper estimates were calculated within four major clades within *Viburnum* (Figure 4; Additional file 2). However, estimates of species discrimination varied dramatically depending on the proportion of the *Viburnum* clade sampled and the method used to implement the barcode (see [9] for further discussion). When we used genetic distances the discrimination rate decreased to 18% (Table 1). Within *Viburnum* subclades we found that none of the average genetic distances were greater than 1%; that is, only one species could be recognized within each of these clades (Figure 4; Additional file 2). Overall, our findings based on the intensive sampling of a single group of plants yields far lower estimates of discriminatory power than the 70% reported in broader surveys using *rbc*L *+ mat*K that include fewer closely related species [5]. As noted above, this result in *Viburnum* does not appear to reflect prevalent hybridization or allopolyploidy.

Supplementary barcodes have been proposed as a means to improve the efficacy of *rbc*L *+ mat*K in discriminating closely related species, especially in groups with low levels of genetic variation [3-5]. We evaluated four additional markers and applied the two most variable, *trn*H-*psb*A and nrITS, as supplementary barcodes, and this yielded some improvement in discrimination. Using a character-based method, we could differentiate up to 98% of *Viburnum* with *rbc*L *+ mat*K + nrITS (Table 1). Discrimination rates using genetic distances were consistently lower (0% at the 2% level with *rbc*L *+ mat*K), and improvement based on the addition of supplementary barcodes depended on the gene region (0.46% and 73% at the 2% level with *rbc*L *+ mat*K + nrITS and *rbc*L *+ mat*K + *trn*H-*psb*A, respectively; Table 1).

Our findings highlight four major points discussed below: (1) for some plant groups, *rbc*L *+ mat*K will not be variable enough to differentiate closely related species; (2) estimates of the discriminatory power of the *rbc*L *+ mat*K barcode have been overestimated by not including demonstrably closely related species; (3) discriminatory success on a regional level depends on the particular representation of subclades within genera within an area; and (4) phylogenetic trees provide the necessary framework for evaluating the success of barcoding as a function of relatedness.

### *rbc*L *+ mat*K rarely differentiate closely related *Viburnum* species

Of the loci we sampled, *mat*K and *rbc*L were the least variable, and the least able to differentiate closely related species. All other loci examined had average genetic distances greater than 1%; *trn*H-*psb*A was the most variable chloroplast locus and nrITS was the most variable marker of those tested (Figure 4). *trn*H-*psb*A was rejected as a core plant barcode because of difficulties in amplification and sequencing [5], and because inverted repeats may also be prevalent [45]. Potential problems with nrITS, including inconsistent amplification and incomplete concerted evolution, have been thoroughly discussed in opposition to the use of nrITS as a core barcode [5,12,46]. Recent work has revisited the use of nrITS, and more specifically ITS2 [30,32,47], due to its universality and ease of amplification from many types of preserved tissues (e.g., old herbarium specimens; processed plants in herbal medicines). Despite potential difficulties, *trn*H-*psb*A and nrITS can be very useful supplementary barcodes within some plant groups [6,12,31,48-51], and this is certainly the case in *Viburnum*.

In future work it will be important to bear in mind potential interaction effects in combining more and less variable markers. Thus, in our case, the core + supplementary barcode was outperformed by the supplementary barcode alone. However, this result is sensitive to the method used to apply the barcode. In character-based methods, adding more markers simply adds more information. In genetic distance approaches, adding highly variable markers to invariable markers dilutes the genetic distances, making species discrimination less likely. *trn*H-*psb*A and nrITS are useful as supplementary barcodes, but may actually be more effective when used alone in groups with slower rates of molecular evolution. Our findings suggest that for species identification purposes alone it may be an inefficient use of time and money to continue to sequence *mat*K and *rbc*L in groups where these markers show very little variation.

*Viburnum* plants are woody (shrubs and small trees) with relatively long generation times and slow rates of molecular evolution as compared to more rapidly evolving herbaceous lineages [52]. We believe that the limited variability seen in *Viburnum* will characterize many other groups of woody plants. Indeed, several studies of woody plant groups are consistent with this prediction regardless of the methods used to assess species discrimination. For example, *rbc*L alone is unable to distinguish genera within Juglandaceae [50], and neither *rbc*L nor *mat*K could discriminate species of *BerberisFicus*, or *Gossypium*[6]. Studies of *Ligustrum* (Oleaceae; [49]) and *Alnus* (Betulaceae; [48]) show that *trn*H-*psb*A and nrITS discriminated two to six times as many species as either *rbc*L or *mat*K. And in *Quercus* (which may have additional complications owing to hybridization) *mat*K and *rbc*L were unable to distinguish any of the 12 sympatric species examined [51]. And, among non-flowering woody plants, the *rbc*L and *mat*K barcode were not variable enough to differentiate Mexican cycads [53] or species of *Picea*[54]. The method of implementing barcodes is not uniform across these studies. However, the message is clear; levels of genetic variation in woody plants are low and barcoding is less successful. Character-based methods may make best use of little variation as these methods could potentially rely on as little as a single base pair [16]. However, it will be important to consider the minimum difference for species identification and to have proper intraspecific sampling to verify the consistency of DNA sequences within a species. Lastly, in woody plant groups where barcoding genes are reported to have higher rates of discrimination [11,55], it would be interesting to establish the phylogenetic relatedness of the species sampled and to increase the species sampling to see if such results continue to hold.

### Insights from sampling closely related species

Our study did not include enough replicates within species to critically compare levels of intra- and interspecific variation. However, given the very low genetic distances, we are confident that the inclusion of more accessions of each species would have very little effect. Instead, an increase in discriminatory power must await the development of more variable markers.

Importantly, we found many cases in which morphologically distinct and geographically separated species were genetically identical or nearly so. For instance, the Mexican species *V. jucundum* and *V. acutifolium* differ dramatically from one another in leaf and inflorescence size [27,56], but are genetically identical according to *rbc*L *+ mat*K. More specifically, *V. jucundum* plants are small trees with leaves averaging 11 cm in length and 9 cm in width, as compared to *V. acutifolium* plants, which are small shrubs with leaves which are typically 4 cm length and 2.5 cm width (i.e., 3x smaller). Genetic distances increased by 0.3% and 0.05% with the addition of *trn*H-*psb*A and nrITS, respectively. Similarly, within the Asian *Succodontotinus* clade, *V. melanocarpum* is readily distinguished by its distinctive black-colored fruits from all of its close relatives with red fruits, yet these species are nearly identical based on the available sequences. For example, the distances separating *V. melanocarpum* from *V. dilatatum* ranged from 0.17-0.55% depending on the supplementary barcode used.

This is not to say, however, that all of the species in our analysis can be easily distinguished based on morphology alone. Species boundaries in *Viburnum* are especially difficult in the Andes of South America (Figure 3; the clade containing *V. ayavacense* through *V. undulatum*; see [57]), where populations have been diverging from one another for only a short time [58,59]. Included in this clade are eight species from Ecuador that are genetically and morphologically quite similar. Although these species cannot be distinguished based on the barcodes examined here, our recent field studies confirm that these are distinct based a combination of one or more morphological characters, on microsatellite data, and on their geographic ranges (Donoghue, Sweeney, and Clement, MS in prep.).

### Species discrimination in a regional context

Community-level or regional barcoding studies are becoming more common, and typically report higher species discrimination rates. In general, this reflects the fact that local floras are mainly comprised of distantly related species, typically representing many families and orders. Success in discriminating species within the genera with two or more species within an area will depend on how closely related these species are, which will vary depending upon speciation mechanisms and the biogeographic history of the group in question. We examined species discrimination in *Viburnum* in two broad regions, which yielded contrasting results.

Japanese *Viburnum* species represent six major clades (*LantanaOpulusPseudotinusSolenotinusSuccodontotinus*, and the isolated *V. urceolatum*), which have long been evolving separately ([15]; Figure 3). Not surprisingly, our discrimination success was quite high in this case. By comparison, 15 of the 17 Mexican and Central American *Viburnum* species are all members of a single major clade, *Oreinodontotinus* (Figure 3), and have radiated into the mountains of this region quite recently [58,59]. Understandably, our success in discriminating the species in this area was very low. The general message is that successful discrimination depends directly on the evolutionary and biogeographic history of the group in question, which can vary dramatically from one community or region to another.

## Conclusions

Our study suggests that broad comparative studies of the success of the proposed plant barcodes have tended to overestimate the discriminatory power by failing to include a sufficient number of comparisons of very closely related species. In particular, the power of the *rbc*L + *mat*K barcode is overrated. In *Viburnum* it is generally possible to confidently distinguish species belonging to the different major clades using the core barcodes, but the failure rate is very high when we consider close relatives within these clades. Even when we are able to differentiate species within these clades using a character-based approach (i.e., accepting any single nucleotide difference), genetic diversity is extremely low and methods based on genetic distances generally fail to distinguish close relatives even when these show clear-cut morphological and geographical differences. We suspect that similar results will be found in other plant groups, but especially in other woody plant groups with relatively long generation times and slow rates of molecular evolution [52]. Moving forward, we encourage the evaluation of the relative success of barcoding in an explicitly phylogenetic context, where the relative relatedness of the species being sampled can be established with confidence. To the extent that our findings are general, we also encourage the plant barcoding community to expand the multilocus barcode to include the additional markers necessary to accurately discriminate between closely related species. Although this may mean compromising somewhat on the ease of amplification and on universality, we believe that the benefits of being able to accurately identify a much higher proportion of species will be well worth the extra effort.

## Authors’ contributions

WLC carried out DNA sequencing and analyses. WLC and MJD designed the study and wrote the manuscript. Both authors read and approved the final manuscript.

## Supplementary Material

Additional file 1 Voucher and Genbank information for *Viburnum* species include in the study, arranged according to major clades (Winkworth and Donoghue, 2005; Clement and Donoghue, 2011). Voucher specimen information includes collector, collector number (No.), and herbarium. Genbank numbers are reported for each gene region; missing data are indicated by a “-.” Herbaria acronyms are as follows: Missouri Botanical Garden (MO), Arnold Arboretum (A), Yale University (YU), New York Botanical Garden (MY), Field Museum (F), University of Washington (WTU), and Kew Royal Botanic Gardens (K). Accessions used in interspecies comparisons are indicated in bold, accesions marked by an asterisk indicate data used in Clement and Donoghue 2011, and accesions marked by a “†” are new to the study of *Viburnum* phylogeny. Click here for file

Additional file 2 Summary of interspecific comparisons for four *Viburnum* clades. The name of each clade is followed by the total number of species described in the group. For each clade, the number of species analyzed, the aligned sequence length, the number of variable characters, the number of unique sequences, and the maximum number of species that can be identified by the data (Max ID rate =Identical sequences/total number of species) are reported. Summary statistics of genetic distances using a Kimura 2-parameter (K2P) model include: minimum genetic distance (Min), maximum genetic distance (Max), mean interspecific distance (Mean) with standard deviation (SD), and the proportion of comparisons of genetic distances greater than 1% (>1%) and greater than 2% (>2%). (PDF 73 kb) Click here for file
