# Supplementary material for: Barcoding success as a function of phylogenetic relatedness in Viburnum, a clade of woody angiosperms
Source: BMC Evol Biol. 2012 May 30;12:73. doi: 10.1186/1471-2148-12-73 (PMC3433329; doi:10.1186/1471-2148-12-73)
Supplement: Additional file 2 — Summary of interspecific comparisons for four Viburnum clades. The name of each clade is followed by the total number of species described in the group. For each clade, the number of species analyzed, the aligned sequence length, the number of variable characters, the number of unique sequences, and the maximum number of species that can be identified by the data (Max ID rate =Identical sequences/total number of species) are reported. Summary statistics of genetic distances using a Kimura 2-parameter (K2P) model include: minimum genetic distance (Min), maximum genetic distance (Max), mean interspecific distance (Mean) with standard deviation (SD), and the proportion of comparisons of genetic distances greater than 1% (>1%) and greater than 2% (>2%). (PDF 73 kb) [file 1471-2148-12-73-S2.pdf]

Additional File 2. Summary of interspecific comparisons for four *Viburnum* clades. The name of each clade is followed by the total number of species described in the group. For each clade, the number of species analyzed, the aligned sequence length, the number of variable characters, the number of unique sequences, and the maximum number of species that can be identified by the data (Max ID rate =Identical sequences/total number of species) are reported. Summary statistics of genetic distances using a Kimura 2-parameter (K2P) model include: minimum genetic distance (Min), maximum genetic distance (Max), mean interspecific distance (Mean) with standard deviation (SD), and the proportion of comparisons of genetic distances greater than 1% (>1%) and greater than 2% (>2%).

Gene Region	Species	Aligned Length	Variable characters	Unique sequences	%Max ID rate	Min	Max	Mean (SD)	> 1%	> 2%
Lantana (15 species)										
*mat*K	10	719	8	4	40.00	0	0.0098	0.0026 (0.0037)	0	0
*rbc*L	10	491	1	2	20.00	0	0.0020	0.0010 (0.0010)	0	0
*rpl*32-*trn*L	9	839	9	7	77.78	0	0.0073	0.0030 (0.0019)	0	0
*trn*H-*psb*A	10	401	4	4	40.00	0	0.0077	0.0040 (0.0028)	0	0
*trn*K	10	1057	4	4	40.00	0	0.0028	0.0008 (0.0008)	0	0
ITS	10	593	34	10	100.00	0.00169	0.0386	0.0214 (0.0121)	71.11	55.56
*rbc*L+*mat*K	10	1210	9	5	50.00	0	0.0066	0.0019 (0.0022)	0	0
*rbc*L+*mat*K+ *trn*H-*psb*A	10	1614	13	8	80.0%	0	0.0069	0.0025 (0.0018)	0	0
*rbc*L+*mat*K+ITS	10	1803	43	10	100.00	0.00056	0.0160	0.0081 (0.0039)	37.78	0
Oreinodontotinus (39 species)										
*mat*K	24	719	6	6	25.00	0	0.0070	0.0014 (0.0015)	0	0
*rbc*L	24	491	2	3	12.50	0	0.0041	0.0010 (0.0013)	0	0
*rpl*32-*trn*L	26	880	13	10 (13)	38.46	0	0.0058	0.0013 (0.0013)	0	0
*trn*H-*psb*A	27	403	15	10 (18)	37.04	0	0.0237	0.0051 (0.0050)	19.66	1.42
*trn*K	22	1057	10	8 (9)	36.36	0	0.0057	0.0018 (0.0013)	0	0
ITS	28	597	49	22 (24)	78.57	0	0.0328	0.0099 (0.0062)	44.97	8.73
*rbc*L+*mat*K	21	1210	8	9	42.86	0	0.0050	0.0013 (0.0010)	0	0
*rbc*L+*mat*K+ *trn*H-*psb*A	22	1612	22	14 (18)	63.63	0	0.0069	0.0022 (0.0016)	0	0
*rbc*L+*mat*K+ITS	22	1805	49	20	90.90	0	0.0106	0.0041 (0.0023)	1.30	0

Gene Region	Species	Aligned Length	Variable characters	Unique sequences	Max ID rate	Min	Max	Mean	> 1%	> 2%
Solenotinus (25 spe										
*mat*K	8	719	5	3	37.50	0	0.0070	0.0017 (0.0021)	0	0
*rbc*L	10	491	1	2	20.00	0	0.0021	0.0004 (0.0008)	0	0
*rpl*32-*trn*L	8	841	9	6	75.00	0	0.0072	0.0032 (0.0019)	0	0
*trn*H-*psb*A	12	429	14	7	58.33	0	0.0213	0.0053 (0.0052)	25.76	1.52
*trn*K	8	1057	6	4	50.00	0	0.0047	0.0016 (0.0016)	0	0
ITS	11	590	45	9	81.82	0	0.0604	0.0168 (0.0170)	60.00	18.18
*rbc*L+*mat*K	7	1210	5	3	42.86	0	0.0041	0.0012 (0.0013)	0	0
*rbc*L+*mat*K+*trn*H-*psb*A	7	1632	14	5	71.43	0	0.0056	0.0021 (0.0017)	0	0
*rbc*L+*mat*K+ITS	7	1800	24	7	100.00	0.0017	0.0078	0.0045 (0.0021)	0	0
Succodontotinus (33 species)										
*mat*K	19	719	5	5	26.32	0	0.0042	0.0010 (0.0009)	0	0
*rbc*L	21	491	5	5	22.73	0	0.0083	0.0029 (0.0026)	0	0
*rpl*32-*trn*L	19	885	15	8	42.11	0	0.0084	0.0033 (0.0027)	0	0
*trn*H-*psb*A	21	394	3	4	19.05	0	0.0077	0.0017 (0.0017)	0	0
*trn*K	19	1068	7	5	26.32	0	0.0047	0.0011 (0.0013)	0	0
ITS	18	605	50	16	88.89	0	0.0369	0.0190 (0.0083)	86.76	48.53
*rbc*L+*mat*K	19	1210	10	9	47.37	0	0.0058	0.0019 (0.0014)	0	0
*rbc*L+*mat*K+*trn*H-*psb*A	18	1604	11	9	50.00	0	0.0057	0.0019 (0.0014)	0	0
*rbc*L+*mat*K+ITS	16	1815	57	16	100.00	0.0006	0.0141	0.0072 (0.0032)	22.86	0
